# EMPLOYMENT AND LIFE SATISFACTION IN LATER LIFE: THE MEDIATING ROLE OF SUBJECTIVE EXPECTATION

**DOI:** 10.1093/geroni/igad104.2159

**Published:** 2023-12-21

**Authors:** Si Young Song, Bomi Choi, Hayoung Park, Miseon Kang

**Affiliations:** Kyung Hee University, Seoul, Republic of Korea; Yonsei University, Seoul, Republic of Korea; Yonsei University, Seoul, Republic of Korea; Yonsei University, Seoul, Republic of Korea

## Abstract

Although the productive aging framework has been emphasized to promote active roles such as employment to improve psychological well-being in later life, the mechanisms that explain the association between employment and life satisfaction remain unclear. Therefore, this study sought to examine the mediation role of subjective expectation about future living standards in the association between employment and life satisfaction among older Korean adults. The data was the 8th wave (2020) of the Korean National Longitudinal Study on Aging, and the sample included 4,238 older Korean adults aged 65 or above. Employment was coded as 0 when they are not working and 1 when working. Life satisfaction was measured by overall quality of life on a ten-point scale. Subjective expectation, the mediating variable, was measured by the subjective evaluation of future living standard on a ten-point scale (e.g., “I think my standard of living will get better in the future”). PROCESS MACRO model 4 in SPSS 26.0 were utilized to test indirect and direct effects while gender, education level, household income, and presence of spouse were controlled for. As a result, employment had a direct effect on life satisfaction and indirect effects were also significant, indicating employment contributes to a higher level of life satisfaction through higher subjective expectation. The findings revealed that the mechanism between employment and life satisfaction among older adults can be explained through subjective expectation, indicating the importance of considering beneficial effects of employment that enhance psychological well-being when developing intervention programs for older adults.

